# Self‐Assembled Bifunctional Peptide as Effective Drug Delivery Vector with Powerful Antitumor Activity

**DOI:** 10.1002/advs.201600285

**Published:** 2017-01-11

**Authors:** Rangrang Fan, Lan Mei, Xiang Gao, Yuelong Wang, Mingli Xiang, Yu Zheng, Aiping Tong, Xiaoning Zhang, Bo Han, Liangxue Zhou, Peng Mi, Chao You, Zhiyong Qian, Yuquan Wei, Gang Guo

**Affiliations:** ^1^ State Key Laboratory of Biotherapy and Cancer CenterDepartment of NeurosurgeryWest China HospitalSichuan UniversityCollaborative Innovation Center for BiotherapyChengdu610041P. R. China; ^2^ Department of Pharmacology and Pharmaceutical SciencesSchool of MedicineTsinghua UniversityCollaborative Innovation Center for BiotherapyBeijing100084P. R. China; ^3^ Key Laboratory of Xinjiang Phytomedicine ResourcesShihezi832002P. R. China

**Keywords:** bifunctional peptides, docetaxel, drug delivery, nanoparticles, self‐assembly

## Abstract

E‐cadherin/catenin complex is crucial for cancer cell migration and invasion. The histidine‐alanine‐valine (HAV) sequence has been shown to inhibit a variety of cadherin‐based functions. In this study, by fusing HAV and the classical tumor‐targeting Arg‐Gly‐Asp (RGD) motif and Asn‐Gly‐Arg (NGR) motif to the apoptosis‐inducing peptide sequence‐AVPIAQK, a bifunctional peptide has been constructed with enhanced tumor targeting and apoptosis effects. This peptide is further processed as a nanoscale vector to encapsulate the hydrophobic drug docetaxel (DOC). Bioimaging analysis shows that peptide nanoparticles can penetrate into xenograft tumor cells with a significantly long retention in tumors and high tumor targeting specificity. In vivo, DOC/peptide NPs are substantially more effective at inhibiting tumor growth and prolonging survival compared with DOC control. Together, the findings of this study suggest that DOC/peptide NPs may have promising applications in pulmonary carcinoma therapy.

## 1 Introduction

Lung cancer is the leading cause of cancer death among males in many parts of the world, and in more developed countries, it has surpassed breast cancer among females.^1^, ^2^ Advanced non‐small‐cell lung cancer (NSCLC) is responsible for most of the death. Docetaxel (DOC), erlotinib, and pemetrexed are the clinically approved second‐line therapies for NSCLC.^3^, ^4^ DOC is a semisynthetic product derived from the needles of the European yew, Taxus baccata. The antineoplastic activities of DOC were played by promoting assembly of tubulin into microtubules, and rendering the microtubules resistant to depolymerization.^5^, ^6^ In response to the increasing challenge of lung cancer worldwide, polymeric nanoparticles (NPs), polymeric micelles, and liposomes have been drawn significant attention to the delivery of DOC to lung cancer cells via a passive targeting mechanism.^7^, ^8^ Although many strategies have been adopted to treat lung cancer, the low target specificity for cancer cells is the most common and serious problem.

NPs have the capability of delivering high doses of therapeutic compounds to tumor cells and have attracted increasing attention in drug delivery and cancer therapy.^9^, ^10^, ^11^ Encapsulation of hydrophobic drugs into NPs can make the drug dispersible in aqueous solutions, which can be used for intravenous applications. The nanoscale and hydrophilic interface of the NPs can prolong their circulation time and enhance the cellular uptake.^12^ Recently, vaccines, peptides, proteins, small molecules, and other biomimetic carriers have been reported for various therapeutic payloads delivery.^13^, ^14^, ^15^ Among these, small molecules or peptides have been developed for active targeting of cytotoxic agents to tumor cells,^16^, ^17^ with the advantage of tissue‐specific targeting, thus off‐target effects could be minimized.^18^, ^19^, ^20^ These vectors with unique structures are able to self‐assemble into a variety of nanostructures including nanocircles, nanotubes, and spherical NPs, which can enhance the gene or drug loading (DL) capability for their high‐aspect‐ratio structures.^21^, ^22^ Moreover, taking the peptides for example, it is easy to endow it with targeting ability through simple modifications, such as conjugated with specific sequences and mediated by avidin–biotin interaction.^16^, ^23^ RGD (Arg‐Gly‐Asp), a tripeptide motif, is an integrin‐recognition motif found in many ligands, allowing for homing of drugs to tumor blood vessels.^24^, ^25^

RGD‐containing peptides have been widely used in tumor‐targeting research.^25^ Peptides containing the Asn‐Gly‐Arg (NGR) motif which can recognize aminopeptidase N (CD13), a membrane‐bound enzyme associated with angiogenic tumor vessels, can be used for delivering various anti‐tumor agents to the tumor vasculature.^26^ Based on this, RGD‐ and NGR‐containing peptides can actively target to tumor blood vessels by binding to integrin and CD13 receptors. Neuronal (N)‐cadherin, a cell adhesion molecule, can mediate cell–cell adhesion via a calcium‐dependent mechanism. Researchers have shown that inappropriate expression of N‐cadherin by tumor cells may promote motility and invasion in carcinoma cells.^27^, ^28^ Histidine‐alanine‐valine (HAV) motif, is a highly conserved sequence at classical cadherin homophilic binding site. It has been confirmed that linear or cyclic peptides containing the HAV sequence of N‐cadherin can disrupt cadherin‐mediated cell adhesion, and hence inhibit cell aggregation, compaction, and neurite outgrowth.^29^, ^30^ In addition, AVPIAQK, Smac/DIABLO (smacN7), is an inhibitor of apoptosis protein antagonist and a promoter of caspase activation.^31^

**Figure 1:**
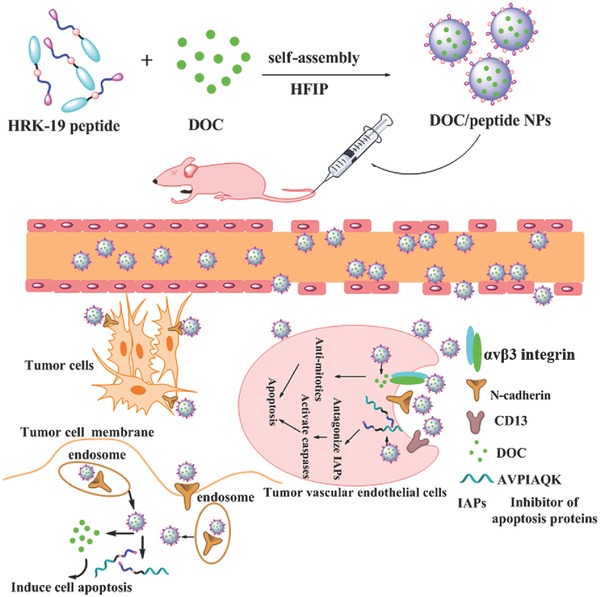
Schematic representation of mechanisms of the DOC/peptide NPs inhibiting tumor growth of mice.

In this study, we have designed a bifunctional peptide HAVRNGRRGDGGAVPIAQK (HRK‐19), conjugated with tumor targeting peptide sequences HAV and NGRRGD, as well as apoptosis‐inducing peptide sequence AVPIAQK. We further prepared self‐assembled NPs using the bifunctional peptide and DOC for potential application in drug delivery systems. The DOC loaded peptide NPs (DOC/peptide NPs) were prepared by directly dissolving in aqueous buffer with DOC and the bifunctional peptide which was pretreated with hexafluoro‐2‐propanol to achieve molecular level mixing. Antitumor effects of the DOC/peptide NPs were investigated both in vitro and in vivo. Molecular‐modeling study was employed to investigate the interactions of DOC and the bifunctional peptide. The cellular uptake and cell apoptosis of the DOC/peptide NPs were also examined. The potential mechanism of the DOC/peptide NPs inhibiting tumor growth in mice is illustrated in the **Figure** **1**: 1) Targeting tumor‐enriched‐cadherin by HAV sequence; 2) Targeting tumor‐enriched‐integrin and ‐CD13 by NGRRGD sequence; 3) Transportation of NPs into tumor cells through endocytosis; 4) Inducing antitumor effects by apoptosis‐inducing peptide AVPIAQK and DOC. Our findings indicated that the DOC/peptide NPs showed significant antitumor activity both in vitro and in vivo, and may present a promising DOC formulation for cancer chemotherapy.

## 2 Results

### 2.1 Tumor‐Targeting Peptide Design and Self‐Assembly Peptide Nanoparticles Preparation

**Figure 2:**
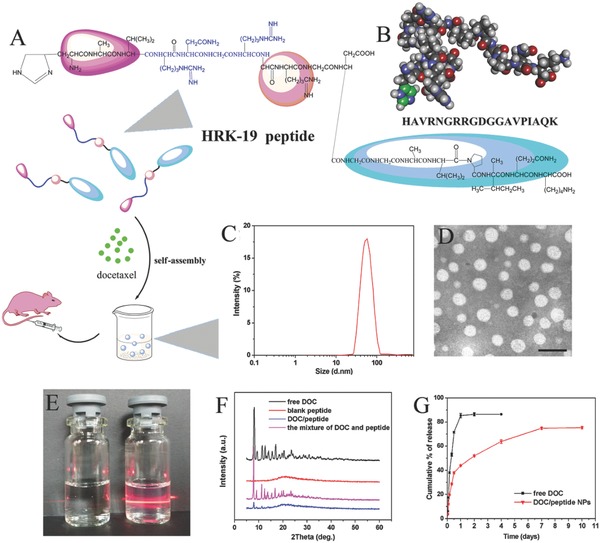
Self‐assembly characterization of the DOC/peptide NPs. A) Schematic illustration of the self‐assembly of DOC/peptide NPs. B) Amino acid sequence and molecular model of the peptide investigated. The carbon atoms on the imidazole ring of histidine residue are colored with green. C) Particle size distribution of DOC/peptide NPs. D) TEM image of DOC/peptide NPs in Dulbecco's phosphate‐buffered saline (DPBS), the scale bar represents 100 nm. E) Photograph of DPBS (a), and DOC/peptide NPs in DPBS (b). F) XRD patterns of docetaxel, peptide, and DOC/peptide NPs. G) In vitro release behavior of DOC from DOC/peptide NPs.

The mass spectrum of the HRK‐19 peptide was shown in Figure S1 of the Supporting Information. And it can be seen the mass of HRK‐19 peptide was 1959.80 g mol^−1^, which was consistent with the theoretical mass (1959.20 g mol^−1^). The HRK‐19 peptide had ≥95% purity, as confirmed by analytical reversed‐phase (RP)‐high performance liquid chromatography (HPLC) (Figure S2, Supporting Information). As shown in Figure S3 of the Supporting Information, the HRK‐19 peptide contains no apparent secondary structure such as helix and Beta turn. As shown HRK‐19 is an amphiphilic peptide with its N‐terminal hydrophilic and C‐terminal hydrophobic (Figure S4, Supporting Information). To prepare a tumor‐targeting peptide, in this study, Asn‐Gly‐Arg‐Arg‐Gly‐Asp (NGRRGD) and three amino acids (HAV) were conjugated with apoptosis‐inducing peptide sequence AVPIAQK, forming bifunctional peptide (HRK‐19 peptide, HAVRNGRRGDGGAVPIAQK). As shown schematically in **Figure** **2**A, self‐assembly of the DOC/peptide NPs was prepared by the film dispersion method. The amino acid sequence and molecular model of the peptide was shown in Figure 2B. The DL, encapsulation efficiency (EE), particle size and polydisperse index (PDI) of a series of DOC/peptide NPs were showed in Table S1 of the Supporting Information. It can be seen that the DL, and particle size increased with increase of drug/copolymer ratio in feed, whereas, the EE decreased accordingly. In consideration of DL and stability, the sample of S2 was chose for further applications and was characterized in detail. The DL and EE of DOC were 4.52 ± 0.18% and 90.50 ± 3.68%. According to the particle size distribution spectrum shown in Figure 2C, the average particle size and PDI of the DOC/peptide NPs were 62.41 ± 0.83 nm, 0.125 ± 0.021, and the zeta potential was 3.07 ± 0.13 mV (Figure S5, Supporting Information). In addition, the prepared NPs were monodisperse and had a very narrow particle size distribution. Transmission electron microscopy (TEM) imaging was carried out to identify the possible nanostructures self‐assembled from the prepared bifunctional peptide. And it can be seen that the peptide NPs observed by TEM (Figure 2D) was in good agreement with the results of particle size analysis. The nanostructure of DOC/peptide NPs observed by particle size analysis, as well as TEM imaging, demonstrated that the prepared DOC/peptide NPs were spherical NPs, and could be well‐dispersed in aqueous solution. Figure 2E showed the appearance of Dulbecco's phosphate‐buffered saline (DPBS), and clear solution with Tyndall effect of DOC/peptide NPs in DPBS (right) could be observed, which revealed that DOC/peptide NPs are stable and could be well‐dispersed in DPBS. Figure 2F presented the X‐ray diffraction (XRD) patterns of DOC, pure peptide, and DOC/peptide NPs. Pure DOC is crystalline, with characteristic peaks at 2θ = 8.10°, 9.32°, 11.38°, 12.60°, 13.94°, 16.94°, and 20.42°. Pure peptide is noncrystalline. When comparing XRD diagrams of DOC, the absence of specific diffraction peaks in the DOC/peptide NPs diagram indicated that DOC was relatively completely encapsulated. In vitro release profile of DOC from DOC/peptide NPs in PBS solution at pH 7.4 was presented in Figure 2G. DOC was rapidly released and reached its peak release of 85.37 ± 1.83% of the total drug within 24 h. In comparison, DOC was released from the DOC/peptide NPs over an extended period.

### 2.2 Interaction between DOC and the HRK‐19 Peptide

**Figure 3:**
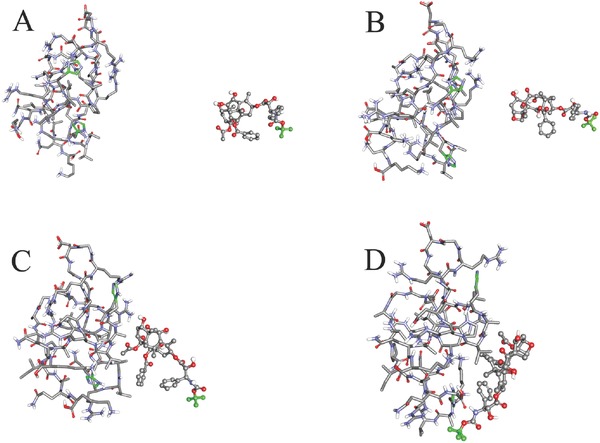
Interaction modes between peptides and DOC revealed by molecular dynamics simulation in water environment. A) The initial conformation of complex composed of two molecules of peptides and docetaxel; Conformations (B)–(D) are corresponding to snapshots of the complex collected at 88.000, 104.250, and 200 ps, respectively. Peptides are represented with stick. The carbon atoms on the imidazole ring of peptides are colored with green. Docetaxel is shown ball‐and‐stick style and the carbon atoms of its terminal methyl group are colored with green.

The interaction between two molecules of HRK‐19 peptide and DOC was investigated with the method of molecular dynamics simulation. At first, DOC was merged randomly to the simulated peptides in the workspace of Hyperchem so as to get the initial structure of a complex. Then two stages of molecular dynamics simulations were performed in the environment of water and solvation effect was considered implicitly. In the heating stage, complex was heated from 0 to 300 K within 100 ps. Subsequently, the complex was running at 300 K for 100 ps. Results of molecular dynamics simulation were presented in **Figure** **3**. It was noted that both the HRK‐19 peptide and DOC were trying to adjusting their conformations and the position until a favorable interaction mode was reached. It can be seen from the overall shape of the HRK‐19 peptide and from the relative positions of the carbon atoms on the imidazole ring of peptides that the conformation of the peptide changed a lot. After 200 ps molecular dynamics simulation, a binding site between the peptide and the DOC can be formed on the surface of the HRK‐19 peptide due to continuous interaction (Figure 3D). Interaction energy between two molecules of peptide and DOC, which was calculated at molecular mechanic level with CHARMM27 force field,^32^ is −38.378 kcal mol^−1^. At the initial state (Figure 3A), the interaction energy between two molecules of peptide and DOC should be close to 0 kcal mol^−1^, and the interaction energy at final state (Figure 3D) was −38.378 kcal mol^−1^. This showed strong interaction between HRK‐19 peptide and DOC.

### 2.3 Cellular Uptake

**Figure 4:**
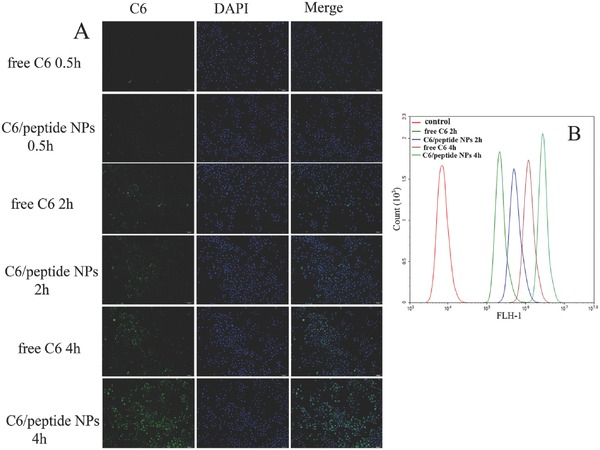
Cellular uptake of free coumarin, coumarin/peptide by A549 cells. A) Confocal images of cells treated with free coumarin and coumarin loaded NPs at 100 ng for 0.5, 2, and 4 h. B) Mean fluorescence intensity of cells after treatment for 0.5, 2, and 4 h. Cells incubated in media only were used as control.

To investigate their ability to transport hydrophobic molecules into cancer cells of the DOC/peptide NPs, cellular uptake studies of drug loaded peptide NPs were performed on A549 cells. Coumarin‐6 (C6) was chosen as the model dye loaded into peptide NPs because of its strong fluorescence and low solubility in water, using the same procedure as for DOC loading.^33^ **Figure** **4**A showed the time‐dependent cellular uptake of free C6, and C6/peptide NPs into A549 cancer cells, evidenced by the stronger green fluorescence in the cells with longer incubation time. In free C6 group, a very dim fluorescence was observed in the cytosol of several cells after 2 h, and fluorescence intensity was increased after incubation for 4 h. In contrast, stronger fluorescence intensity can be found for C6/peptide NPs group than free C6 after 0.5 h. C6/peptide NPs could rapidly accumulate in the A549 cells in 2 h, and a more bright green fluorescence was observed after 4 h. These results were further confirmed quantitatively by flow cytometry (FCM) measurements (Figure 4B), again revealing a consistent increase in fluorescence intensity when the incubation time changed from 0.5 to 4 h. And it also showed that fluorescence intensity of cells treated with C6/peptide NPs is much stronger than that in free C6 group at the same incubation time.

### 2.4 The Effects of DOC on Microtubule Formation

**Figure 5:**
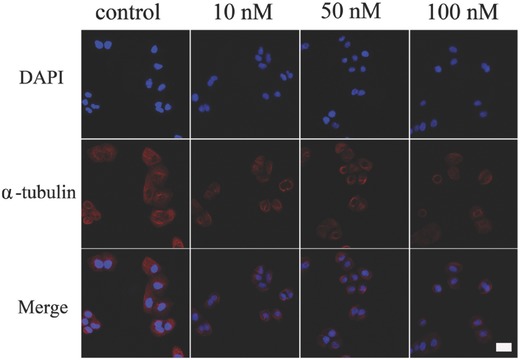
The effects of DOC on the microtubule formation on the A549 cell lines. A549 cells were seeded on coverslip and treated with docetaxel of indicated concentrations for 4 h. The microtubule was determined by fluorescence microscopy. The red indicates the α‐tubulin and the blue is the DAPI stain for DNA. Scale bar: 20 µm.

Agents targeting microtubules are often used in cancer therapy. The taxanes act to stabilize microtubules and dampen microtubule dynamics to prevent the normal formation of mitotic spindles.^34^ Both paclitaxel and DOC can bind to β‐tubulin in assembled tubulin, thereby reducing depolymerization and leading to cell death.^35^ Therefore, we examined the effects of DOC on microtubule formation and cell apoptosis on A549 cells. **Figure** **5** showed the microtubule formation examined by fluorescence microscopy. Microtubule formation was assayed by the tubulin stain (red) and the nuclear was revealed by DAPI (4′,6‐diamidino‐2‐phenylindole) stain (blue). As shown in Figure 5, in the absence of DOC, cells go through the cell cycle with the formation of normal tubulin spindle in (Figure 5, Control). However, at 10 × 10^−9^ m, DOC begins to arrest A549 cells by interfering with the assembling/disassembling microtubule spindle.

### 2.5 Cell Apoptosis Assay

**Figure 6:**
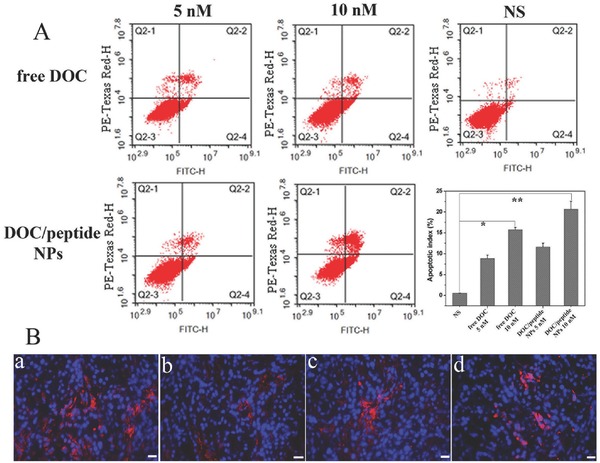
Cell apoptosis assay in vitro and *N*‐cadherin immunoreactivity in vivo. A) Ratio of apoptotic and necrotic cells treated for 24 h with various DOC formulations. The asterisks “*”and “**” on graph obtained by Student's *t*‐test indicate significant differences at *P* < 0.05 and *P* < 0.01, respectively. B) Photomicrograph shows strong membrane positivity (the red) of *N*‐cadherin in the tumor cells: a) normal saline group, b) blank peptide group, c) Taxotere group, and d) DOC/peptide NPs group.

Given the effectiveness of transporting hydrophobic drug‐DOC into A549 cancer cells by the bifunctional peptide NPs, we carried out the cytotoxicity experiments to further evaluate the anticancer activity of DOC/peptide NPs on A549 by Flow cytometry analysis. Flow cytometry analysis was performed to monitor the percentage of cell apoptosis after the cells being stained by Annexin V‐Fluorescein isothiocyanate/propidium iodide (Annexin V‐FITC/PI) apoptosis detection kit, and A549 cells incubated in media only were used as control (**Figure** **6**A). The percentage of apoptosis of A549 cells (early and late apoptosis) incubated for 24 h with DOC/peptide containing DOC 5 × 10^−9^ and 10 × 10^−9^ m were 11.56 ± 0.91% and 20.66 ± 1.89%, respectively. For free DOC, they were 8.87 ± 0.79% and 15.73 ± 0.56%, respectively. Obviously, DOC‐loaded bifunctional peptide NPs induced significant cell apoptosis. In short, these results indicated that the bifunctional peptide NPs could facilitate the inhibition of tumor proliferation and induction of tumor cell apoptosis, as was consistent with the results concerning cellular internalization described above, leading to stronger anti‐tumor efficacy.

### 2.6 In Vivo Therapeutic Efficacy

**Figure 7:**
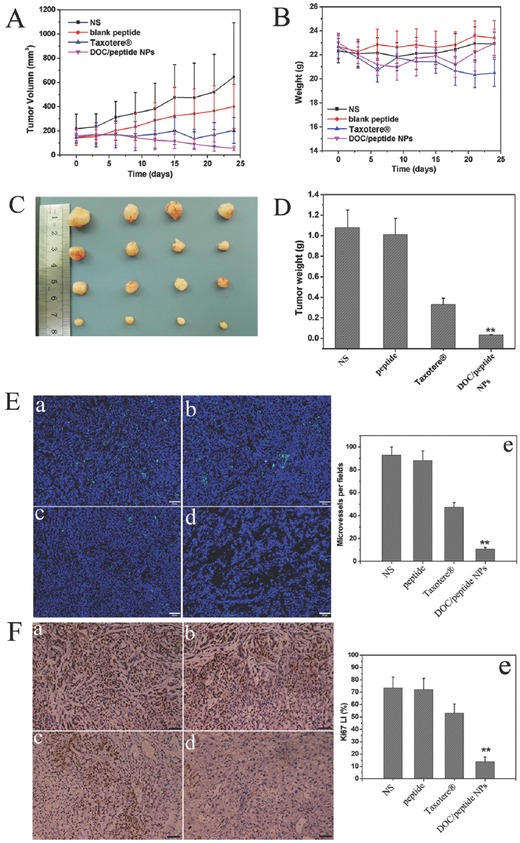
DOC/peptide NPs inhibited growth in subcutaneous A549 model. A) Tumor development curve. Balb/c nude mice were subcutaneously with 100 µL of A549 cells on day 0. On day 7, the mice were randomly assigned to four groups that received intravenous normal saline (NS), blank peptide, Taxotere, or DOC/peptide NPs. B) The body weights of different groups. C) Representative photos of tumors in each treatment group. D) The tumor weights of the mice measured on the indicated days. E) CD31 immunofluorescent staining of tumors. F) Ki67 immunohistochemical staining of tumors. The asterisk “**” on graph obtained by Student's *t*‐test indicates significant differences at *P* < 0.01. In panels (D) and (E), the four groups were a) NS group, b) blank peptide group, c) Taxotere group, and d) DOC/peptide NPs group, respectively.

Subcutaneous A549 model and pulmonary metastatic A549 model were used to compare the antitumor activity of DOC/peptide NPs with that of Taxotere, blank peptide, and normal saline (NS). Figure 6B showed the strong membrane positivity of N‐cadherin in the tumor cells. Cells treated with the free peptide can induce partial loss of cadherin labeling. And treatment with DOC/peptide NPs showed more loss of cadherin labeling than free peptide. As shown in **Figure** **7**A, fast tumor growth curves were obtained in the control groups (NS and blank peptide), suggesting that the A549 tumor growth was not inhibited by blank peptide. Compared with the NS groups, DOC/peptide NPs was effective in inhibiting tumor growth (Figure 7A–D). As shown in Figure 7B, Taxotere treatment induced a decrease in the body weight of the mice. According to Figure 7D, tumor weight in DOC/peptide group (0.035 ± 0.005 g) is significantly lower than that in Taxotere (0.3325 ± 0.060g, *P* < 0.001), blank peptide (1.01 ± 0.159 g, *P* < 0.001), or NS group (1.08 ± 0.17 g, *P* < 0.001). This further confirmed that DOC/peptide exhibited better therapeutic efficacy than Taxotere in A549 tumor‐bearing mouse model (Figure 7D).

Angiogenesis is a process vital to the continued development of a tumor mass. This process has been the subject of intense research due to its role in cancer development.^36^ Sections of tumors from mice in each group were stained for CD31 immunofluorescence to determine the microvessel density (MVD) as a measurement of tumor angiogenesis. The DOC/peptide NPs treatment resulted in dramatic inhibition of angiogenesis in the tumors (Figure 7E). The MVD in the DOC/peptide NPs group was 10.67 ± 1.53, which was dramatically lower than that in the Taxotere group (47.33 ± 4.04, *P* < 0.01), blank peptide group (88.00 ± 8.54, *P* < 0.01), and NS (92.67 ± 7.23, *P* < 0.01) group. The results implied that antiangiogenesis may be another mechanism of inhibiting cancer by the DOC/peptide NPs in vivo. Moreover, the activities of various drug formulations on proliferation of tumor cells were analyzed by immune‐histochemical staining for Ki‐67, a proliferation marker (Figure 7F). Compared with NS group (73.50 ± 4.11%, *P* < 0.01), blank peptide group (72.00 ± 9.24%, *P* < 0.01), and Taxotere group (53.00 ± 7.42%, *P* < 0.01), the percentage of Ki‐67‐positive cells in the DOC/peptide NPs‐treated group (13.67 ± 4.11%) was significantly lower.

**Figure 8:**
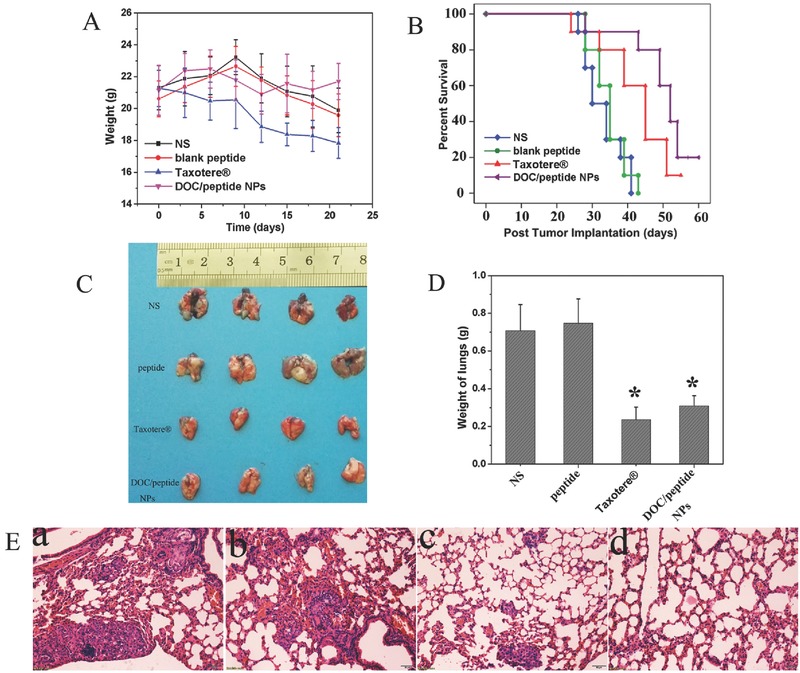
DOC/peptide NPs inhibited growth and metastasis in pulmonary metastatic A549 model. A) The body weights of the mice measured on the indicated days. Balb/c nude mice were injected intravenously with 100 µL of cell suspension containing 1 × 10^7^ A549 cells on day 0. On day 5, tumor bearing mice were assigned randomly into four groups that received intravenous normal saline (NS), blank peptide, Taxotere, or DOC/peptide NPs. B) Kaplan–Meier survival curve of mice in each group. C) Representative photos of lungs in each treatment group. D) Weight of lungs in each group. E) H&E staining of lungs in each treatment group: a) NS group, b) blank peptide group, c) Taxotere group, and d) DOC/peptide NPs group. The asterisk “*” on graph obtained by Student's *t*‐test indicates significant differences at *P* < 0.05.

In pulmonary metastatic model (**Figure** **8**), the body weight of the Taxotere treated animals was significantly lower than that of other groups (Figure 8A). As shown in Figure 8B, the median survival in DOC/peptide group (52 days) is significantly longer compared with Taxotere (45 d, *P* < 0.05), blank peptide (35 d, *P* < 0.05), and NS (30 d, *P* < 0.05) group. The weight of lungs (Figure 8D) in DOC/peptide NPs group (0.31 ± 0.05 g) was dramatically decreased compared with that in blank peptide (0.74 ± 0.13 g, *P* < 0.001), or NS group (0.71 ± 0.14 g, *P* < 0.001). However, the weight of lungs in Taxotere group (0.23 ± 0.07) was lower than that of DOC/peptide NPs group, that was because of the toxic effect of Taxotere on the mice. The H&E staining of lungs showed the anticancer effect of Taxotere group was worse than that of DOC/peptide NPs group. There has obvious tumor in the lungs of the Taxotere group (Figure 8E).

### 2.7 Evaluation of Tumor Targeting and Penetrating Efficiencies of Drug/Peptide NPs in Vivo

**Figure 9:**
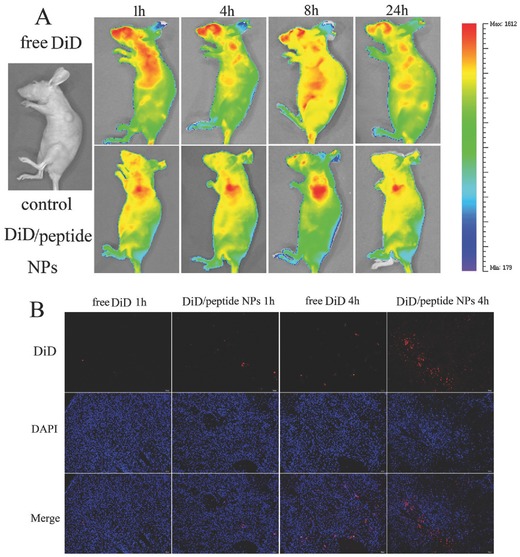
Evaluation of tumor targeting and penetrating efficiencies of drug/peptide NPs in vivo. Mice with forelimb tumors derived from A549 cells were given tail vein injections of NPs loaded with the fluorescent dye DiD (100 µg kg^−1^). Normal saline was served as the control. A) In vivo images of mice after treatment with free DiD or DiD/peptide NPs for 1, 4, 8, and 24 h. B) Frozen sections of tumors removed 1 or 4 h after treatment with free DiD or DiD/peptide NPs were stained with DAPI to label nuclei.

**Figure** **9**A showed the real time distribution and tumor accumulation of physiological saline (NS), free 1, 1′‐dioctadecyl‐3, 3, 3′, 3′‐tetramethyl indotricarbocyanine (DiD), DiD‐loaded bifunctional peptide NPs (DiD/peptide NPs), in the presence of excess free bifunctional peptide (DiD/peptide NPs) at 1, 4, 8, and 24 h after iv injection. After 1 h, high DiD fluorescence was observed in mice, especially in the tumor of mice treated with DiD/peptide NPs (Figure 9A). As time elapsed, the fluorescence accumulated in the tumors of DiD/peptide NPs mice, while the average free DiD signal decreased significantly (Figure 9A). This observation demonstrated that the active targeting group exhibited slower clearance, that is, it showed the long circulation effect of bifunctional peptide NPs systems, which was consistent with cellular uptake results.

To further investigate the accumulation and penetration of DiD/peptide NPs, nude mice bearing tumors from A549 cells were injected with free DiD, DiD/peptide NPs, at a DiD concentration of 100 µg kg^−1^. After treatment for 1 or 4 h, tumors were excised for examination. As shown in Figure 9B, the fluorescence of tumor cells treated with DiD/peptide NPs was higher than free DiD at different time, which was congruent with the in vivo imaging data.

## 3 Discussion

In this study, a type of amphiphilic peptide with tumor‐targeting NGRRGD sequence and HAV sequence and tumor apoptosis‐inducing AVPIAQK sequence were designed and synthesized. The proof‐of‐principle experiments were conducted to test the cancer targeting value of the bifunctional peptide and the therapeutic value of the self‐assembly of DOC/peptide NPs. The results showed that the DOC/peptide NPs were effective in suppressing growth of A549 NSCLC in vitro and in vivo. DOC, a chemotherapeutic agent with a large spectrum of antitumor activity, was approved for second‐line treatment of advanced NSCLC.^37^, ^38^ Due to the poor water solubility, nonionic surfactants—Cremophor EL and Tween 80—were often used for their commercial formulations. The side effects with these nonionic surfactants include hypersensitivity reactions and peripheral neuropathy in human.^39^

Many strategies have been used to improve its water solubility, among these, nanotechnology shows promising application in drug delivery system. The common NPs' delivery of cytotoxic cargoes to tumor tissues can be achieved by passive targeting, which makes the use of the inherent size of NPs and exploits the enhanced permeability and retention (EPR) effect.^40^, ^41^, ^42^ However, the presence of the complex in vivo environment limits the EPR effect, thereby resulting in the poor efficacy in drug delivery. A feasible approach for overcoming this issue is to modify these carriers with different targeting ligands that facilitate the translocation across the endothelium of the tumor vasculature.^43^ NGRRGD can mediate tumor‐homing by binding to α_v_β_3_ and CD13 receptors, which are tumor angiogenesis biomarkers up‐regulated on various cells in tumors.^44^

HAV is a sequence resides in the first extracellular repeat of the classical cadherins. Studies have shown that peptides mimetic the HAV motif in N‐cadherin are sufficient to inhibit cell–cell adhesion and neurite outgrowth.^[26]^ The self‐assembled peptides have been widely investigated because of their good biocompatibility and used for cancer treatment, tissue engineering, and wound healing.^45^, ^46^, ^47^, ^48^ In this study, a bifunctional peptide conjugated with tumor targeting peptide sequences‐NGRRGD and HAV, and apoptosis‐inducing peptide sequence‐AVPIAQK was prepared. Then, we used this bifunctional peptide and a film dispersion method to obtain DOC nanoparticles (DOC/peptide NPs) with a very narrow particle size distribution, thus leading to remarkable improvement in solubility of the DOC in DPBS solution. In addition, the results of particle size distribution, combined with nanostructure of the DOC/peptide NPs observed by TEM, suggested that the prepared NPs were stable and could be well‐dispersed in aqueous solutions. In comparison with free DOC, a much slower release behavior of DOC/peptide NPs can be seen. Cellular uptake of peptide NPs was evaluated using confocal microscopy and FCM, respectively. The results indicated that the improved cytotoxicity of peptide NPs was attributed to their enhanced uptake by cells.

Sugahara et al. showed that co‐administration of iRGD with cancer drugs was slightly more effective than the conjugated drug at inhibiting tumor growth and accumulation.^49^ And Zhao et al. also showed that combined application of α‐helical peptides and antitumor drug doxorubicin at low concentrations was significantly more effective than either drug alone against HeLa tumors.^50^ So in this study, we think coadministration of the bifunctional peptide loaded with DOC appears to have more effective anticancer activity and great clinical potential. The main mechanism of action of DOC against cancer cells is to stabilize microtubules and prevent depolymerization stabilized microtubules that could further lead to cell apoptosis. In cytotoxicity tests, we found that cells treated with free DOC or DOC/peptide NPs exhibited similar concentration‐dependent cell apoptosis and blank peptide had a very low toxicity on tumor cells. These experiments suggest a similar antitumor activity between DOC/peptide NPs and free DOC. Furthermore, in vivo animal experiments showed that the DOC/peptide NPs efficiently inhibited both growth and metastasis of tumors, and prolonged the survival of tumor‐bearing mice in pulmonary metastatic A549 model, all results further confirmed that DOC/peptide NPs exhibited better therapeutic efficacy than commercial formulation Taxotere under the same experiment conditions.

Particles with a small size (<200 nm) can easily extravasate to extravascular tumor site due to the EPR effect.^51^ On the other hand, the bifunctional peptide used in this study can improve its tumor tissue‐specific targeting due to tumor‐targeting sequences—NGRRGD and HAV. Therefore, DOC/peptide NPs may enhance drug accumulation in tumor tissues, and decrease drug extravasation from normal vessels into normal tissues. To confirm above hypothesis, we examined in vivo tumor targeting of peptide NPs with DiD as a fluorescent probe, and we found that DiD/peptide NPs accumulated at higher concentrations in the tumor than free DiD. Moreover, the in vivo antitumor study further demonstrated the superior antitumor activity of DOC/peptide NPs. Results of CD31 staining of tumor tissues suggested improved antiangiogenesis effect of DOC/peptide NPs.

## 4 Conclusions

We report here the use of the bifunctional peptide as a molecular building unit to construct a nanoscale vector to encapsulate the hydrophobic drug DOC. After DOC was encapsulated into peptide NPs, cellular uptake and in vitro cytotoxicity effect of DOC were increased compared with free DOC. Besides, a sustained in vitro release behavior was observed in DOC/peptide NPs group. Bioimaging analysis showed that DOC/peptide NPs could penetrate into xenograft tumor cells with a significantly long retention in tumors and high tumor targeting specificity. In vivo xenograft studies showed that DOC/peptide NPs were more effective in inhibiting tumor growth and prolonged survival. Thus, the DOC/peptide NPs prepared in this work showed improved antitumor activity in vitro and in vivo, and may have potential applications as an intravenous therapy in lung cancer therapy.

## 5 Experimental Section

*Materials*: The HRK‐19 peptide (HAVRNGRRGDGGAVPIAQK, theoretical mass = 1959.20 g mol^−1^, 99.5% purified powder) was synthesized commercially using solid phase synthesis methods by the Shanghai Bootech BioScience & Technology Co., Ltd., Shanghai, China. Peptide stock solutions were prepared by dissolving the peptide powders in DPBS to a concentration of 5 mg mL^−1^, mixing, sonicating for 40 s, filtering, and then storing at 4 °C. For the TEM measurement, the peptide stock solution was diluted to a concentration of 0.25 mg mL^−1^ (100 × 10^−6^ m). DOC was purchased from Sichuan Xieli Pharmaceutical Co., Ltd., (Chengdu, China). Taxotere was commercially available from Sanofi (Paris, France). Annexin V‐FITC Apoptosis Detection Kit was obtained from KeyGen Biotech (Nanjing, China), Ki‐67 Rabbit Monoclonal Antibody was obtained from Abcam (Massachusetts, USA). DAPI, C6, and 3‐(4, 5‐dimethyl‐2‐thiazolyl)‐2, 5‐diphenyl‐2H‐tetrazolium bromide (methyl thiazolyl tetrazolium) were obtained from Sigma‐Aldrich (Saint Louis, USA). DiD was purchased from Biotium (Hayward, CA). Dulbecco's modified Eagle's medium and fetal bovine serum (FBS) were purchased from HyClone (Logan, USA). All the animals were provided with standard laboratory chow and tap water ad libitum and all the procedures were performed according to the protocol approved by the Institutional Animal Care and Treatment Committee of Sichuan University (Chengdu, People's Republic of China). All mice were treated humanely throughout the experimental period.

*HRK‐19 Peptide Analysis*: RP‐HPLC of the peptide was performed on an Agilent 1100 liquid Chromatograph with a vydac C18 column (4.6 × 250 mm^2^, 5 mm). Samples performed with 0.1% trifluoroacetic acid (TFA) in aqueous solution/0.1% TFA in ACN (gradient elution from 80:20 to 10:90, v/v) over 20 min, and then run using gradient elution from 10:90 to 0:100 (v/v) for another 5 min, followed by a plateau to complete 30 min.

The mass spectrometric analysis was carried out using the LCQ Deca XP mass spectrometer of Thermo Finnigan (San Jose, CA, USA) in the ESI (+) (electrospray ionization) mode. The peptide solution was introduced into the ESI source by direct injection using a Thermo syringe pump at a flow rate of 0.5–1 µL min^−1^. Spray voltage was maintained at ≈5.0 kV, the capillary voltage was about 15 V, and the capillary temperature was set at 250 °C, N_2_ was used as a nebulizing gas.

Secondary structure analysis was performed by using GOR IV online tools (https://npsa‐prabi.ibcp.fr/cgi‐bin/npsa_automat.pl?page = npsa_gor4.html). And hydrophobicity/hydrophilicity analysis was performed by using ProtScale online tools (http://web.expasy.org/protscale/) based on Kyte & Doolittle.

*Preparation and Characterization of Self‐Assembly Peptide Nanoparticles*: The drug encapsulation experiments were performed as follows: DOC and the appropriate bifunctional peptide were dissolved in 400 µL of hexafluoro‐2‐propanol (HFIP) in 5 mL glass vials and sonicated for 1 min to mix, before the HFIP was removed by rotary evaporation. The vials were then left to stand in the fume hood for at least 8 h at room temperature with the cap removed to allow any trace amount of HFIP to evaporate. Then 200 µL of 1 × DPBS was added to each vial and vortexed for 30 s. The solutions were aged for 8 h and then centrifuged (2000 g, 5 min) to remove any precipitated DOC, and the supernatant was carefully collected and analyzed using HPLC with the following conditions: Varian ProStar model 325 HPLC (Agilent Technologies, Santa Clara, CA, USA) equipped with an Agilent Zorbax‐C18 column (5 µm, 4.6 × 150 mm); the flow rate was 1 mL min^−1^, with the mobile phase held at 35% A (MeCN with 0.1%TFA) and 65% B (0.1%TFA aqueous solution) for 5 min and start to gradient to 70% A at a period of 25 min, then gradient back to the initial conditions in 1 min and held for 4 min; the monitored wavelength was 237 nm. The drug EE was calculated as the percentage of DOC recovered from the supernatant to the DOC added. The DL capacity was calculated from the percentage of recovered DOC to the sum of recovered DOC and added bifunctional peptide.

The particle size distribution, PDI, and zeta potential of prepared DOC/peptide NPs were determined by Malvern Nano‐ZS 90 laser particle size analyzer at 25 °C. All results were the mean of three test runs, and all data were expressed as the mean ± standard deviation (SD). The morphological characteristics of the self‐assembly peptide NPs were examined by transmission electron microscope (TEM, H‐6009IV, Hitachi, Japan). The NPs were diluted with distilled water and placed on a copper grid covered with nitrocellulose. Samples were negatively stained with phosphotungstic acid and dried at room temperature.

*The 3D Structures of DOC/Peptide from Computer Simulations*: At first, the structure of the DOC molecule was built by using Marvin Sketch (http://www.chemaxon.com) and optimized at a molecular mechanical level using the MMFF94 method.^52^ Then, it was further optimized at semi‐empirical level using the AM1 method with the Fletcher‐Reeves algorithm by employing Hyperchem software (HyperChem Professional 8.0, Hypercube, Inc., Gainesville, FL, USA). In order to understand in detail the interaction between the HRK‐19 peptide and DOC, 200 ps molecular dynamics simulation was performed on complex composed DOC and two molecules of the peptide. In the process of simulation, CHARMM27 was chosen as the force field and solvation effect was considered implicitly.^32^

*Cell Culture, Cellular Uptake, and Cell Apoptosis Assay*: The human NSCLC cell line A549 were purchased from American Type Culture Collection and cultured with RPMI‐1640 (GIBCO,US), supplemented with 10% (V/V) FBS (GIBCO, US), 100 unit mL^−1^ penicillin, and 100 µg mL^−1^ streptomycin. Cellular uptake of C6 as a model drug loaded in self‐assembly peptide NPs was measured by confocal microscopy and flow cytometry analysis. A549 cells at log phase were seeded onto a 24‐well plate at 1 × 10^5^ cells per well and cultured in 1 mL of medium. After 24 h, the media was removed, and cells were exposed to serum‐free medium containing free C6, or C6/peptide NPs at a final concentration of 10 µg mL^−1^. After incubation for 0.5, 2, and 4 h, the media were removed and carefully washed with PBS. For observation by confocal microscopy, cells were fixed with cold acetone, washed again with PBS, stained with DAPI, and imaged using a fluorescence microscope (×400) (Leica DM2500, Germany).

For the expression and localization of α‐tubulin, cells were incubated with the antibodies against α‐tubulin (Abcam, USA) followed by incubation with TRITC‐conjugated secondary antibodies. The images were obtained using a confocal microscope (DM6000 CS, Leica, Germany).

To investigate the apoptotic effect of the DOC/peptide NPs, flow cytometry was performed using an ESP Elite flow cytometer (BeckmaneCoulter, Miami, FL, USA) with FITC‐conjugated Annexin V/propidium iodide (PI, BD PharMingen) staining as per the manufacturer's instructions. A549 cells cultured in 6‐well plates were treated with DOC/peptide NPs, free DOC, and blank peptide. Medium without treatment reagents was added as control. Both early apoptotic (Annexin V‐positive, PI‐negative) and late apoptotic (Annexin V‐positive and PI‐positive) cells were measured.

*In Vivo Tumor Targeting and Penetrating Efficiencies*: For the in vivo animal experiments, tumor‐bearing female nude BALB/c mice were injected intravenously with free DiD or an equivalent amount of DiD (100 µg kg^−1^) loaded in the drug/peptide NPs. After i.v. injection of the drug, optical fluorescence imaging was performed by positioning each mouse on an animal plate in the vivo imaging system (Quick View 3000) with excitation and emission wavelengths of 645 and 715 nm, respectively. The images were obtained 1, 4, 8, and 24 h after injection of the test drugs. For the in vivo cellular uptake studies, BALB/c nude mice bearing subcutaneous tumors from A549 cells were injected with free DiD and DiD/peptide NPs. After 1 and 4 h, the tumors were isolated and embedded in optimal cutting temperature compound, and then cut into 5 µm slices. Sections were stained by DAPI and observed.

*In Vivo Tumor Model and Treat Plant*: Antitumor activity of DOC/peptide NPs was investigated in pulmonary metastatic A549 model and subcutaneous A549 model. Eight‐week‐old female BALB/c mice nude were obtained from the Animal Center Laboratory of Beijing HFK Bioscience Co., Ltd. In subcutaneous A549 model, the mice were injected subcutaneously with 100 µL of an A549 cell suspension (1 × 10^7^) into the right flank. After the tumor mean diameter reached ≈6 mm, the tumor‐bearing mice were randomly assigned to four groups that received NS (control), blank peptide, commercial formulation Taxotere (Sanofi, Paris, France) (5 mg kg^−1^ body weight), or DOC/peptide NPs (5 mg kg^−1^ body weight) by intravenous injection into the tail three times for 3 weeks. When the mice in the control group began to die, all mice were sacrificed by cervical vertebra dislocation, and the tumors were immediately harvested and measured. To further investigate the antitumor activity of DOC/peptide NPs in subcutaneous A549 model, survival times of the mice were observed (10 mice per group).

In pulmonary metastatic A549 model, BALB/c nude mice were injected intravenously with 100 mL of cell suspension containing 2 × 10^5^ A549 cells on day 0. On day 5, tumor bearing mice were assigned randomly into four groups (12 mice per group). Mice were injected intravenously every 3 d for 2 weeks with 100 µL of NS (control), blank peptide, commercial formulation Taxotere (Sanofi, Paris, France) (5 mg kg^−1^ body weight), or DOC/peptide NPs (5 mg kg^−1^ body weight), respectively. For tumor growth inhibition assay (6 mice per group), mice were scarified by cervical verte bradislocation on day 25. On day 26, mice in NS group began to die. Lungs in each group were weighted, and tumor nodules in each lung were numbered. To further study the antitumor activity of DOC/peptide in pulmonary metastatic A549 model, survival times of the mice were observed (6 mice per group).

*Immunohistochemistry*: Tumor tissue sections were prepared as described above for Ki‐67 staining using the labeled streptavidin‐biotin method.^53^ The primary antibody and secondary antibody were rat antimouse monoclonal antibody Ki‐67 (Gene Tech) and biotinylated goat antirat immunoglobulin (BD Biosciences Pharmingen), respectively. To quantify Ki‐67 expression, the Ki‐67 labeling index (Ki‐67 LI) was calculated as number of Ki‐67 positive cells/total number of cells counted under×400 magnification in five randomly selected areas in each tumor sample by two independent investigators in a blinded fashion.

For the CD31 assay, the tumors were stored at −80 °C to examine microvessel expression, then frozen sections of tumors were cut at 8–10 µm thickness using a cryostat (Leica Microsystems, CH), fixed in acetone, and washed with PBS. After permeabilization (Triton X‐100 (Sigma‐Aldrich, DE) 0.1% (v/v) in PBS) and blocking (5% (w/v) bovine serum albumin (BSA) in PBS), the primary antibody (rat anti‐CD‐31 (1:50), BD Pharmingen, USA) was applied for 24 h at 4 °C, and followed by incubation with an FITC‐conjugated second antibody (Abcam, USA). Finally, sections were incubated with DAPI (Invitrogen, BE) (50 ng mL^−1^) for 5 min to visualize the cell nuclei. MVD was calculated as the average number of small CD31‐positive vessels in a high‐power (×400) field using a fluorescence microscope (×400) (Leica DM2500, Germany). The immunofluorescent analysis of N‐cadherin was evaluated as above. Briefly, the primary antibody (monoclonal antihuman N‐cadherin (1:200), Abcam, USA) was applied for 1 h at 37 °C, and followed by incubation with Alexa Fluor 647‐conjugated second antibody (Abcam, USA). Finally, sections were incubated with DAPI (Invitrogen, BE) (50 ng mL^−1^) for 5 min to visualize the cell nuclei.

*Statistical Analysis*: Statistical analyses were performed using SPSS for Windows version 15.0 (IBM Corporation, Armonk, NY, USA). Data were presented as means ± SD/SEM. Statistical analysis was performed using two‐tailed Student's *t*‐test, and *P*‐value <0.05 was considered as significant difference.

## Supporting information

As a service to our authors and readers, this journal provides supporting information supplied by the authors. Such materials are peer reviewed and may be re‐organized for online delivery, but are not copy‐edited or typeset. Technical support issues arising from supporting information (other than missing files) should be addressed to the authors.

Supplementary
