# Supplementary material for: Self‐Assembled Bifunctional Peptide as Effective Drug Delivery Vector with Powerful Antitumor Activity
Source: Adv Sci (Weinh). 2017 Jan 11;4(4):1600285. doi: 10.1002/advs.201600285 (PMC5396162; doi:10.1002/advs.201600285)
Supplement: Supplementary file 1 — Supplementary [file ADVS-4-na-s001.pdf]

Copyright WILEY-VCH Verlag GmbH & Co. KGaA, 69469 Weinheim, Germany, 2016.

ADVANCED SCIENCE
Open Access

Supporting Information

for *Adv. Sci.,* DOI: 10.1002/advs.201600285

Self-Assembled Bifunctional Peptide as Effective Drug Delivery Vector with Powerful Antitumor Activity

Rangrang Fan, Lan Mei, Xiang Gao, Yuelong Wang, Mingli Xiang, Yu Zheng, Aiping Tong, Xiaoning Zhang, Bo Han, Liangxue Zhou, Peng Mi, Chao You, Zhiyong Qian, Yuquan Wei, and Gang Guo**

supplementary information

Self-Assembled Bi-Functional Peptide as Effective Drug Delivery Vector with Powerful Anti-Tumor Activity

Rangrang Fan[1,†], Lan Mei[1,†], Xiang Gao[1], Yuelong Wang[1], Mingli Xiang[1], Yu Zheng[1], Aiping Tong[1,*], Xiaoning Zhang[2], Bo Han[3], Liangxue Zhou[1], Peng Mi[1], Chao You[1], Zhiyong Qian[1], Yuquan Wei[1], Gang Guo[1,*]

[1] State Key Laboratory of Biotherapy and Cancer Center, and Department of Neurosurgery, West China Hospital, Sichuan University, and Collaborative Innovation Center for Biotherapy, Chengdu, 610041, PR China

[2] Department of Pharmacology and Pharmaceutical Sciences, School of Medicine, Tsinghua University, and Collaborative Innovation Center for Biotherapy, Beijing 100084, P. R. China

[3] Key Laboratory of Xinjiang Phytomedicine Resources, Shihezi 832002, PR China

[*] Corresponding author: Gang Guo, E-mail: guogang@scu.edu.cn (G. Guo), aipingtong@scu.edu.cn (A.P.Tong). Tel: (86) 028-8516 4063, Fax: +86 28 85164060.

[†] These authors contributed equally to this work.

Supplementary Table and Figure

Table S1. The characterization of DOC/peptide NPs.

Sample	DOC:Peptide	DL (%)	EE (%)	Size (nm)	PDI
S1	2.5%	2.35 ± 0.07	94.13 ± 2.95	58.01 ± 1.17	0.177 ± 0.015
S2	5%	4.52 ± 0.18	90.50 ± 3.68	62.41 ± 0.83	0.125 ± 0.021
S3	8%	6.32 ± 0.21	79.08 ± 2.62	66.27 ± 5.82	0.29 ± 0.035

Abbreviations: DL, Drug loading; EE, Encapsulation efficiency.

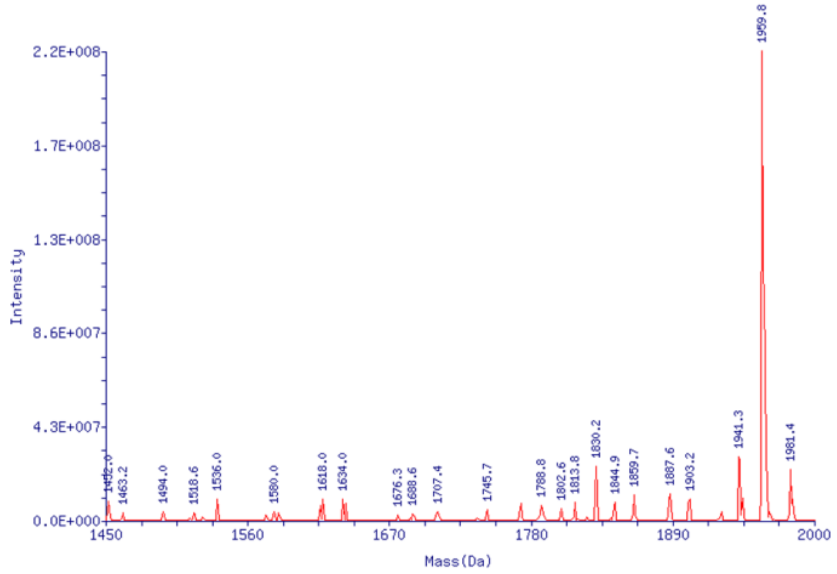

Figure S1. The mass spectrum of the HRK-19 peptide.

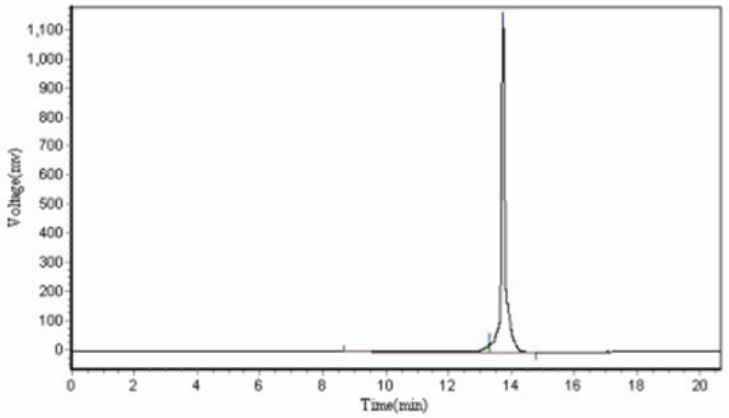

Results

Peak No.	Peak ID	Ret Time	Height	Area	Conc.
1		13.303	27371.934	415983.750	3.8983
2		13.722	1127351.875	10254883.000	96.1017
Total			1154723.809	10670866.750	100.0000

Figure S2. The RP-HPLC spectrum of the HRK-19 peptide.

GOR4 :

Alpha helix	(Hh)	:	0	is	0.00%
3_{10} helix	(Gg)	:	0	is	0.00%
Pi helix	(Ii)	:	0	is	0.00%
Beta bridge	(Bb)	:	0	is	0.00%
Extended strand	(Ee)	:	8	is	42.11%
Beta turn	(Tt)	:	0	is	0.00%
Bend region	(Ss)	:	0	is	0.00%
Random coil	(Cc)	:	11	is	57.89%
Ambiguous states (?)		:	0	is	0.00%
Other states		:	0	is	0.00%

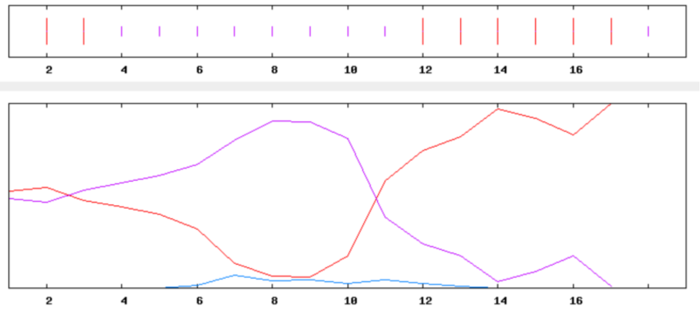

Figure S3. Secondary structure analysis of HRK-19 peptide.

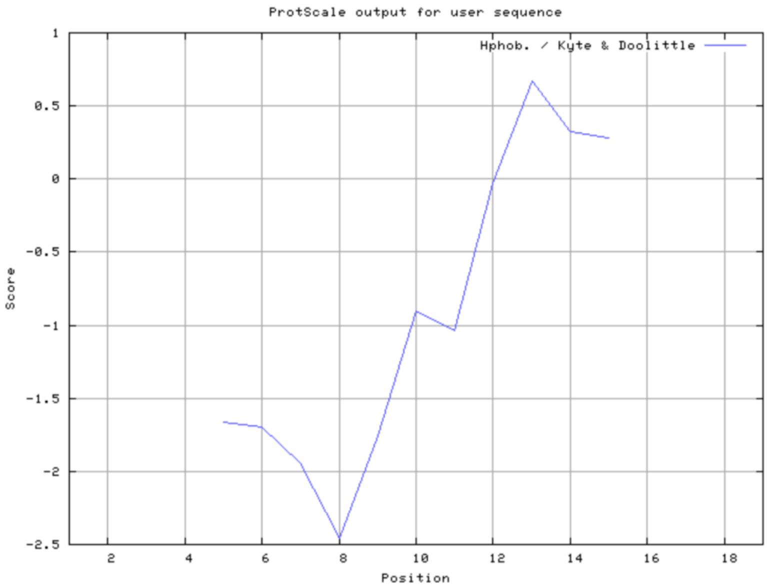

Figure S4. Hydrophobicity/Hydrophilicity analysis of HRK-19 peptide.

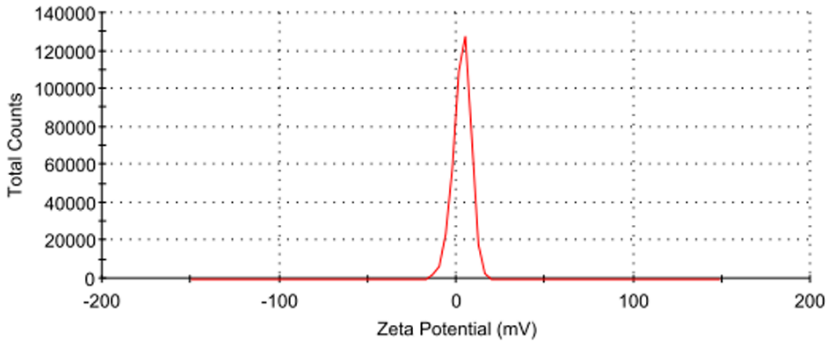

Figure S5. The zeta potential of the DOC/peptide NPs.
